# Cluster-level convergence of malaria infection, childhood zero-dose prevalence and health-campaign frequency in sub-Saharan Africa: a Bayesian analysis of 18 countries

**DOI:** 10.1136/bmjgh-2026-025607

**Published:** 2026-11-11

**Authors:** Victor Alegana, Bridget Farham, Franck Mboussou, Dorothy Achu, Benido Impouma

**Affiliations:** 1 World Health Organization Regional Office for Africa, Brazzaville, Congo; 2 World Health Organization, Country Office, the Democratic Republic of the Congo, Kinshasa, Congo (the Democratic Republic of the)

**Keywords:** Malaria, Immunisation, Vaccines, Nutrition

## Abstract

**Introduction:**

Health campaigns reach populations that routine services miss yet remain planned and delivered vertically by disease programmes. Zero-dose children, who have received no dose of a diphtheria-tetanus-pertussis containing vaccine, concentrate in many settings that also carry high malaria transmission. The spatial relationship between the burdens campaigns target and campaign frequency has not been quantified below national and subnational scales.

**Methods:**

Demographic and health surveys and Malaria Indicator Surveys from 18 sub-Saharan African countries (2015–2025) provided geo-located survey clusters. A three-level Bayesian hierarchical model (survey cluster, first subnational administrative unit (Admin-1), country) was fitted to malaria rapid diagnostic test (RDT) positivity in children aged 6–59 months and zero-dose prevalence in children aged 12–23 months. Admin-1 estimates were ranked into tertiles; units highest for both indicators were classified as carrying converging burden. Campaigns recorded for 2019–2025 were scored on start-time proximity, matching geographic scope and documented co-delivery; pairs starting within 8 weeks and scoring above a pre-set threshold were treated as compatible for combined delivery.

**Results:**

Cluster-level correlation between RDT positivity and zero-dose prevalence was positive in 12 of 18 countries, strongest in Mozambique (posterior mean 0.43, 95% credible interval 0.33 to 0.52). Of 220 Admin-1 units, 113 simultaneously carried elevated malaria prevalence, zero-dose prevalence and insecticide-treated net access–use gap, concentrated in the Democratic Republic of the Congo (24), Angola (17) and Côte d’Ivoire (11); 84 (74%) fell in the highest tertile of campaign frequency. Of 1456 eligible campaigns, 1216 pairs targeting different intervention domains started within 8 weeks in the same country; 338 (28%) were compatible for combined delivery yet delivered separately.

**Conclusion:**

Malaria infection and childhood zero-dose prevalence converge at cluster and Admin-1 level, yet compatible, temporally proximate campaigns are delivered separately. This concentration identifies where assessing integration would carry most value, but feasibility depends on context-specific constraints.

**WHAT IS ALREADY KNOWN ON THIS TOPIC**

- Health campaigns for malaria, immunisation, neglected tropical diseases and nutrition reach hundreds of millions of children annually in sub-Saharan Africa, yet are largely organised and delivered vertically.
- Malaria infection, zero-dose prevalence and undernutrition are known to cluster geographically at country and large subnational scales, but cluster-resolution evidence of within-country co-occurrence and its alignment with the operational campaign calendar has not been reported.

**WHAT THIS STUDY ADDS**

- A three-level Bayesian hierarchical model fitted to paired survey clusters across 18 sub-Saharan African countries quantifies cluster-level co-occurrence of malaria infection and childhood zero-dose prevalence and links the resulting Admin-1 convergence classification to a 2019–2025 calendar of 1456 health campaigns.
- Of 220 Admin-1 units, 113 carry simultaneously elevated malaria prevalence, zero-dose prevalence and insecticide-treated net access–use gap; 84 of these (74%) fall in the highest tertile of campaign frequency, and 338 cross-domain campaign pairs scored as highly compatible for co-delivery were delivered as separate operations.

**HOW THIS STUDY MIGHT AFFECT RESEARCH, PRACTICE OR POLICY**

- The cluster-resolution convergence map identifies the specific Admin-1 units where assessing the integration of multiple campaigns would be most valuable, while the missed-opportunity analysis identifies the country and disease-domain pairs for which prospective feasibility evaluations of campaign integration would be most justified.
- Country-led feasibility evaluations alongside digital tools to support combined delivery are required to convert the spatial co-occurrence and missed-opportunity signal into a measurable advantage in coverage or cost.

## Introduction

 The diseases addressed by health campaigns across sub-Saharan Africa have geographic overlap, share similar last-mile delivery pathways, and target a similar age-profile of children, yet the campaigns themselves are largely organised vertically.^1–4^ The Africa region accounted for approximately 94% of global malaria cases and 95% of malaria deaths in 2024, with children under five and pregnant women at greatest risk.^5^ An estimated 7.7 million African children, close to 20% of zero-dose children globally, received no dose of a diphtheria-tetanus-pertussis (DTP)-containing vaccine in 2024.^6–8^ More than 350 million people require annual preventive chemotherapy for neglected tropical diseases.^9^ These burdens are not spatially independent since the communities that carry high malaria transmission frequently carry the highest concentrations of under-vaccinated children.

Two decades of disease investment have built large campaign platforms. Insecticide-treated net distribution has delivered over 2.5 billion nets to sub-Saharan Africa since 2004.^10^ Seasonal malaria chemoprevention reached 53 million children across 19 countries in 2023, up from fewer than one million in 2012. Supplementary immunisation activities for measles have reached hundreds of millions of children. Campaign frequency is high and between 2019 and 2025 Nigeria delivered on average more than 20 campaigns per year and Ethiopia 14, the majority as stand-alone events.^11 12^ The documented operational consequences of these campaigns include diversion of primary-care staff, disruption of facility services during peak campaign weeks, fragmented supply chains and persistent geographies of missed children.^13–16^ The spatial relationship between the burdens that campaigns target has been described at national and first sub-national scale, but not at the resolution of the communities in which campaigns are delivered. A policy case for systematic integration of these campaign platforms has recently been articulated, drawing on pilot experience from Ethiopia, Nigeria and the Democratic Republic of the Congo.^17^

Here, the spatial co-occurrence of malaria infection and childhood zero-dose prevalence was quantified directly from survey clusters using a three-level Bayesian hierarchical model, and the resulting Admin-1 estimates were combined into a burden-convergence classification. Campaign scheduling records for 2019–2025 were analysed to quantify how frequently campaigns of different disease domains occur concurrently in the same country and how often temporally close, compatible pairs are nonetheless delivered separately. The analysis covers 18 countries with sufficient paired survey clusters for the correlation estimates and all Admin-1 units of the region with cluster data for the convergence mapping. The contribution is a cluster-resolution estimate of where multiple burdens converge and an empirical characterisation of the integration opportunity that the current campaign calendar presents. Estimates are reported at Admin-1 level, the finest level at which the demographic and health surveys (DHS) sampling frame is designed to be representative.

## Methods

### Scope and data sources

Data were drawn from the most recent DHS and malaria indicator surveys (MIS) for 2015–2025, using the most recent available round per country. Geo-referenced cluster records were extracted for malaria rapid diagnostic test (RDT) results in children aged 6–59 months, receipt of the first and third doses of DTP-containing vaccine (DTP1, DTP3) in children aged 12–23 months, and household insecticide-treated net (ITN) access and use. Zero-dose status was defined based on DTP1 coverage.^18^ Admin-1 boundaries from the DHS spatial repository, merged to a continental layer, provided the basis for spatial analysis. Campaign scheduling and co-delivery records for 2019–2025 were obtained from the Campaign Hub (Linksbridge).^19^ Under-five population denominators were taken from the United Nations World Population Prospects 2024 revision.^20^ No ethical approval was required since all data were drawn from publicly available survey programmes, programmatic databases and global surveillance systems. The analysis workflow utilising both data streams, from source through the exclusions applied at each stage to the analytical outputs, together with the three levels of the spatial hierarchy, is shown in figure 1.

**Figure 1:**
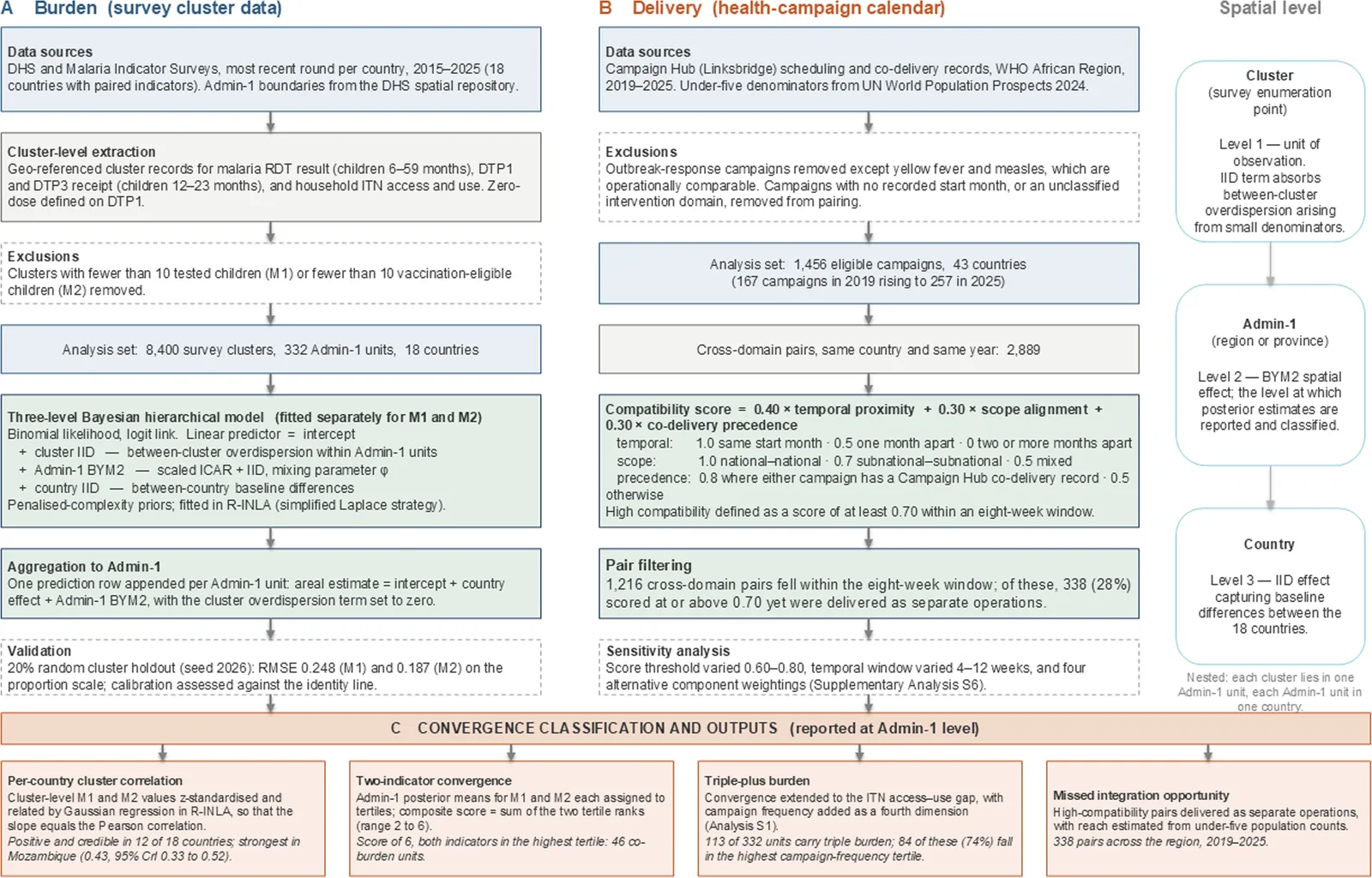
Analysis flow for burden and campaign delivery. BYM2, Besag-York-Mollie type 2; Crl, credible interval; DHS, demographic and health surveys; ICAR, intrinsic conditional autoregressive; IID, independent and identically distributed; ITN, insecticide-treated net; M1, malaria rapid diagnostic test positivity, children 6–59 months; M2, zero-dose prevalence (no DTP1), children 12–23 months; RDT, rapid diagnostic test; RMSE, root mean squared error.

### Cluster-level three-level hierarchical model

Two indicators were modelled independently, the RDT positivity (M1) and zero-dose prevalence (M2) with a binomial likelihood and logit link, taking the survey cluster as the unit of observation. For cluster i in Admin-1 unit j and country k, the linear predictor comprised an intercept and three random effects. These included a cluster-level independent and identically distributed (IID) term capturing between-cluster overdispersion within Admin-1 units; an Admin-1 BYM2 term combining a scaled intrinsic conditional autoregressive (ICAR) spatially structured component and an IID unstructured component, with mixing parameter φ governing the proportion of variance attributable to spatial structure,^21^ and a country-level IID term capturing between-country baseline differences. Cluster counts were taken to be conditionally independent binomial draws given the random effects, with residual heterogeneity between clusters in the same Admin-1 unit absorbed by the cluster-level IID term. The ICAR component was scaled to a generalised variance of one and adjusted for disconnected graph components. Clusters with fewer than ten tested children (M1) or fewer than ten vaccination-eligible children (M2) were excluded. Penalised-complexity priors^22^ were specified for the hyperparameters, anchored at the null model of no random variation. For the BYM2 mixing parameter, P(φ<0.5); and for the BYM2, country and cluster precisions, P(σ>1) equal to 0.01 on the marginal standard-deviation scale (formal model construction in online supplemental analysis S1).^23^ The penalised-complexity prior on the BYM2 mixing parameter was specified as P(φ<0.5)=2/3, and the priors on the BYM2, country and cluster precisions as P(σ>1)=0.01 on the marginal standard-deviation scale. The two indicators were fitted independently rather than jointly, so that any correlation between them is estimated from the data rather than induced by a shared latent field. No covariates were included since the estimates are intended as an empirical description of observed burden rather than a prediction from covariates or correlates.

Models were fitted in R−INLA using the simplified Laplace integration strategy.^24^ Admin-1 posterior means were obtained by appending one prediction row per Admin-1 unit to the cluster stack, so that the areal estimate reflects the intercept, the country effect and the Admin-1 BYM2 effect with the cluster overdispersion term set to zero. The Admin-1 estimate is the model expectation for a cluster of typical composition in that unit, obtained with the overdispersion term set to zero, and is therefore a modelled cluster-level expectation rather than a population-weighted areal mean. Surveys were treated as comparable across rounds within 2015–2025, with no temporal term.

### Burden convergence classification

Admin-1 posterior means for M1 and M2 were each assigned to tertiles, with tertile rank 1 the lowest third and rank 3 the highest. A composite two-indicator convergence score was formed by summing the two tertile ranks (range 2–6) and units with both indicators in the highest tertile (score 6) were classified as the co-burden tier. A three-indicator score reported in online supplemental analysis S2 adds the tertile rank of the ITN access–use gap, giving a range of 3 to 9, and a four-dimensional score adds the tertile rank of the number of campaigns conducted in the unit between 2019 and 2025, giving a range of 4 to 12; units in the highest tertile on all four dimensions are termed triple-plus burden. The ITN access–use gap is the difference, in percentage points, between the survey-weighted Admin-1 proportion of households owning at least one ITN and the survey-weighted proportion of children under five who slept under an ITN the previous night. The ITN gap and campaign frequency were observed Admin-1 quantities rather than modelled posteriors. The former quantity is a difference of two household-level proportions for which the DHS sampling frame is designed to be representative at Admin-1 level. Units with no posterior estimate for an indicator were left unclassified.

### Model validation

Predictive performance was assessed on a 20% random holdout of clusters per indicator using the same prediction-row mechanism, model structure and priors as the main fit. Root mean squared error was computed between the posterior mean and the observed cluster proportion on the held-out clusters, and calibration was examined against the identity line.

### Per-country cluster correlation

For each country with sufficient clusters, cluster-level zero-dose and RDT-positivity values were standardised to zero mean and unit variance and related by a Gaussian linear regression in R-INLA; under standardisation the slope equals the Pearson correlation coefficient. Posterior means and 95% Bayesian credible intervals of the slope were extracted, and a country was reported as a credible positive (or negative) association where the interval excluded zero. The full specification and the per-country estimates are provided in online supplemental analysis S5, online supplemental tables 1a,b.

### Campaign scheduling and compatibility scoring

For each pair of campaigns in the same country and year, a compatibility score was derived from three components: temporal proximity (1.0 if start months were identical, 0.5 if one calendar month apart and 0 if two or more months apart), geographic-scope alignment (1.0 for two national-scope campaigns, 0.7 for two subnational campaigns and 0.5 for mixed scope) and documented co-delivery precedence (0.8 if either campaign had a co-delivery record in the Campaign Hub and 0.5 otherwise), weighted 0.40, 0.30 and 0.30 respectively.


Score=0.40×temporal+0.30×scope+0.30×precedence

Only pairs targeting different primary intervention domains were eligible, the five domains being malaria ITN distribution; malaria indoor residual spraying and seasonal chemoprevention; nutrition; neglected tropical diseases; and immunisation. Polio campaigns were classified within immunisation for the main analysis and treated as a separate sixth domain in online supplemental analysis S2(b), reflecting the distinct scheduling and logistics pathways of national and subnational immunisation days. Pairs with a score of at least 0.70 occurring within an 8-week window were classified as high-compatibility co-delivery opportunities; the count of such pairs delivered separately was reported as the missed opportunity and missed reach, the number of children under five living in the countries in which such a pair was delivered separately, was estimated from under-five population counts. Missed reach indicates the population scale of those pairs rather than a count of children who missed an intervention.

The components follow the shared-planning and calendar-alignment strategy set out in the campaign effectiveness landscape analysis prepared for the Task Force for Global Health, which identified the absence of a framework for assessing coordination opportunities across campaign domains as a priority data gap.^25^ The weights used here represent a stated operational prior rather than an estimated quantity. Temporal proximity carries the largest weight because two campaigns cannot share a microplan, a transport round or a single community-mobilisation event unless they fall in the same period, whereas scope alignment and documented precedence facilitate co-delivery without being necessary for it. Co-delivery is the highest tier of integration (ie, campaigns can be partially integrated without achieving full co-delivery and no empirical data existed to test levels of partial integration). The score takes 15 distinct values, and 0.70 is the value attained by the weakest same-month configuration, two campaigns starting in the same calendar month with mixed geographic scope and no documented co-delivery precedent (ie, 0.40×1.0+0.30×0.5+0.30×0.5=0.70). A threshold of 0.70 therefore includes every same-month pair together with pairs 1 month apart that are national in scope for both campaigns and carry a co-delivery record (0.74). Because the Campaign Hub records a start month rather than a start date, the calendar month is the finest temporal resolution available, and the temporal component falls to zero at a gap of two calendar months, so that the highest score attainable beyond 1 month is 0.54 and no pair separated by more than 1 month can reach 0.70. The 8 week window therefore fixes the denominator against which high-compatibility pairs are expressed rather than the set of pairs identified. Sensitivity of the count to the threshold, the window and the component weights is reported in online supplemental analysis S6 (online supplemental tables 6–9; online supplemental figure 5).

### Supplemental subnational and national analyses

The analyses summarised above are described in full in the online supplemental information, together with additional analyses that extend them. These are an Admin-1 three-indicator convergence classification incorporating the ITN access–use gap and campaign frequency (online supplemental analysis S1); national missed co-delivery opportunities, with a sensitivity analysis treating polio as a distinct domain (online supplemental analysis S2); the spatial variability of DTP1-to-DTP3 dropout (online supplemental analysis S3; online supplemental table 5); a BYM2 regression relating the ITN access–use gap and DTP dropout to zero-dose prevalence (online supplemental analysis S4); and the sensitivity of the compatibility score to its threshold, its temporal window and its component weights (online supplemental analysis S6; online supplemental tables 6–9; online supplemental figure 5); and the per-country cluster-level correlation underlying the co-occurrence result (online supplemental analysis S5; online supplemental tables 1a,b). Online supplemental analysis S1, S3 and S4 operate at Admin-1 resolution and online supplemental analysis S2 at national resolution; they support, rather than constitute, the primary cluster-level result.

## Results

### Cluster-level co-occurrence of malaria and zero-dose

Across 18 countries with sufficient paired survey clusters (8396 clusters in total), the within-country correlation between cluster-level malaria RDT positivity and zero-dose prevalence was positive in 12 countries. The association was strongest in Mozambique (posterior mean 0.43, 95% CrI 0.33 to 0.52), Benin (0.41, 0.32 to 0.49), Senegal (0.39, 0.30 to 0.49) and Nigeria (0.38, 0.32 to 0.43), and was also positive in Madagascar, the Democratic Republic of the Congo, Angola, Gabon, Cameroon, Côte d’Ivoire, the United Republic of Tanzania and Ghana. These results suggest that, in this sample, malaria transmission and zero-dose occur in the same geographies within countries or spatially co-overlap at Admin-1 levels (online supplemental tables 1a,b). The credible interval included zero in the remaining six countries, which were therefore not reported as showing co-occurrence.

Within-country variation in DTP1-to-DTP3 dropout was widest in Mali (coefficient of variation 0.49), Ethiopia (0.46), Kenya (0.42) and Mozambique (0.41), and mean dropout was highest in the Democratic Republic of the Congo (0.49), Angola and Guinea (0.44 each); across the 27 countries assessed the median coefficient of variation was 0.22 (online supplemental analysis S3; online supplemental table 5; online supplemental figure 1). Zero-dose prevalence and DTP dropout were strongly positively associated across 343 Admin-1 units (Pearson correlation 0.82), indicating that units retaining children who never start the vaccination schedule also retain children who start but do not complete it (online supplemental analysis S4; online supplemental figure 2).

The three-level model partitioned variance across the cluster, Admin-1 and country scales (online supplemental analysis S1; online supplemental table 2). The Admin-1 BYM2 mixing parameter φ was 0.998 (95% CrI 0.996 to 1.000) for RDT positivity and 0.789 (0.596–0.913) for zero-dose prevalence, indicating that between-Admin-1 variation in malaria infection was almost entirely spatially structured whereas zero-dose retained a slightly larger unstructured component. The marginal distribution of this parameter is shown in online supplemental figure 3. The mixing parameter is sharply identified for malaria but not for zero-dose prevalence. On the logit scale, the Admin-1 term accounted for 63% of the total latent variance in RDT positivity, while the cluster and country terms accounted for 23% and 14%, respectively. In the case of zero-dose prevalence, these terms were 27% for Admin-1, 24% for cluster, and 49% for country. Subnational spatial structure therefore dominated malaria infection, whereas between-country differences dominated under-vaccination.

### Admin-1 convergence classification

The posterior mean estimates for the two indicators (shown in figure 2A,B) were combined into a convergence classification based on tertiles (figure 2C), resulting in a composite score ranging from 2 to 6. Out of the 342 Admin-1 units for which estimates were available for both indicators, 46 units fell into the highest posterior tertile for both malaria and zero-dose prevalence. These co-burden units were primarily located in the Democratic Republic of the Congo (15 units), Côte d'Ivoire (9 units), Benin and Mozambique (5 units each), Nigeria (4 units) and Angola (3 units), with additional units in Cameroon, Ethiopia, Gabon and Kenya.

**Figure 2:**
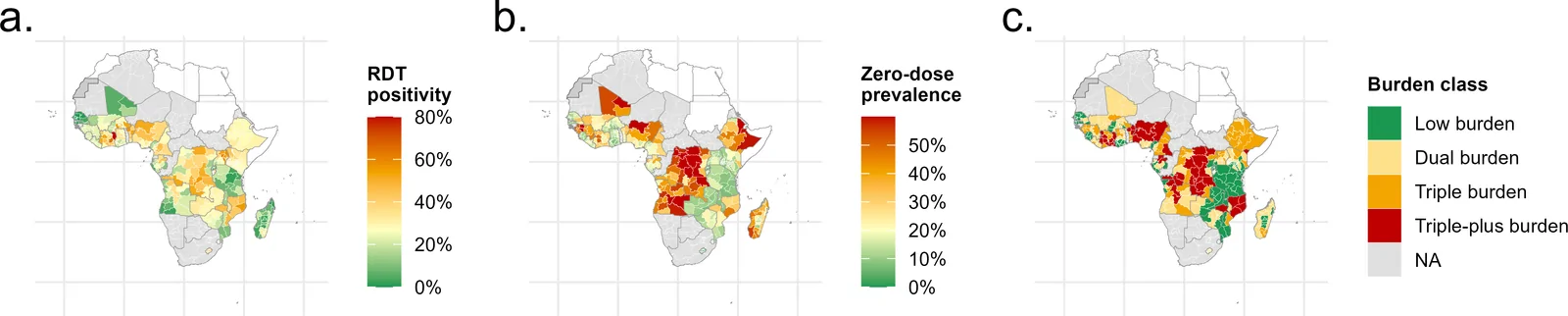
Cluster-derived co-burden of malaria and childhood zero-dose at Admin-1 level. Posterior mean estimates from a three-level binomial BYM2 model fitted to DHS/MIS survey clusters. (A) Malaria rapid diagnostic test (RDT) positivity (children 6–59 months). (B) Zero-dose prevalence (children 12–23 months). (C) Tertile-based co-burden classification (composite score=tertile rank of a+tertile rank of b, range 2–6).

Additionally, 250 Admin-1 units across Africa did not have any survey clusters that met the threshold for inclusion for one or both indicators. This was either because the country had no DHS or MIS conducted within the study window or because the sampled clusters within those units fell below the required ten-child threshold. Consequently, no values were predicted for these units, and they were left unclassified. The cluster-level correlations for each country are presented in figure 3.

**Figure 3:**
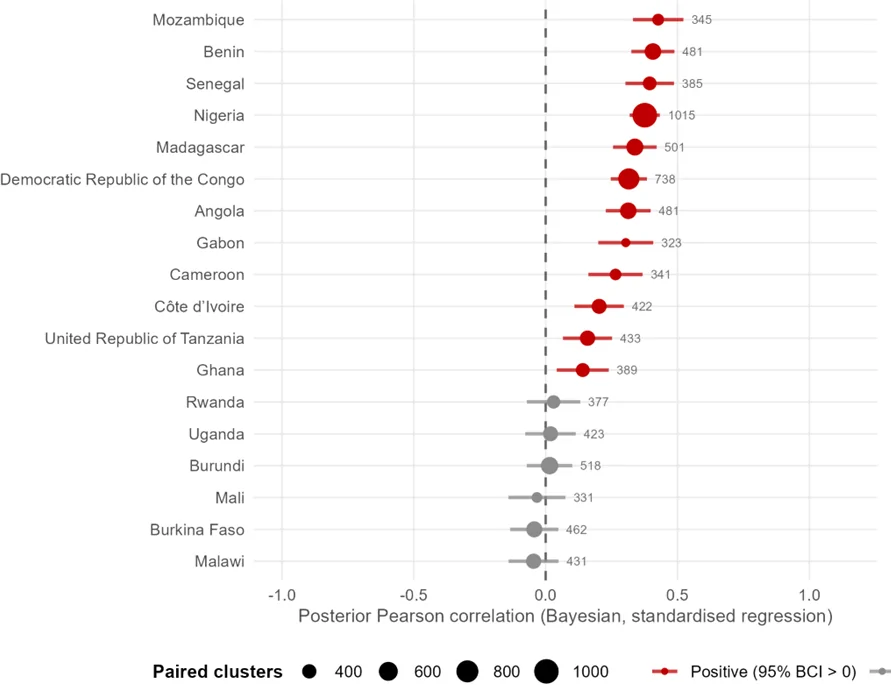
Within-country cluster-level correlation between malaria rapid diagnostic test (RDT) positivity and zero-dose prevalence. Posterior mean (point) and 95% Bayesian credible interval (BCI) (line) of the standardised-regression slope, equivalent to the Pearson correlation, estimated per country from paired survey clusters. Point size is proportional to the number of paired clusters. Countries are ordered by posterior mean; coloured points indicate intervals excluding zero. The dashed line marks zero correlation.

At the Admin-1 level, the median width of the 95% credible interval was 0.81 for RDT positivity and 0.42 for zero-dose prevalence. This difference reflects the smaller sample sizes underlying the malaria indicator at the cluster level. On drawing 4000 replicates from each Admin-1 posterior and recomputing the tertile cut-points for each draw, the average number of units in the co-burden tier was 40, with a 95% interval ranging from 32 to 47, compared with the 46 units identified in the posterior means. Among the 46 units, the median probability of retaining co-burden status was 0.44 (with an IQR of 0.34 to 0.54), and no unit outside of the 46 exceeded a probability of 0.50.

This indicates that the size of the co-burden set and its outer boundary are stable, while the classification of individual units near a tertile cut-point may vary. Since the replicates were drawn independently across units, they disregarded the posterior correlation caused by shared country and spatial terms, which may have led to an overestimation of reclassification. The classification ranks represent where the assessment of integration would be most valuable, but they do not designate specific units.

Predictive performance was assessed on a 20% random cluster holdout. Posterior mean predictions against observed cluster proportions gave a root mean squared error of 0.248 for RDT positivity and 0.187 for zero-dose prevalence on the proportion scale (online supplemental figure 6). Posterior SD in the Admin-1 estimates was greatest in units with few clusters (online supplemental figure 6b).

### Triple-plus burden: convergence of disease and campaign frequency

Extending the convergence framework to the gap between household ITN access and ITN use at Admin-1 level, and to campaign frequency, puts the cluster-level co-occurrence within the operational context (online supplemental analysis S1). At the Admin-1 level, 113 units carried elevated malaria prevalence, zero-dose prevalence and ITN access–use gap simultaneously, concentrated in the Democratic Republic of the Congo (24 units), Angola (17), Côte d’Ivoire (11), Burkina Faso (10) and Mozambique (9). Of these 113 triple-burden units, 84 (74%) fell in the highest tertile of campaign frequency and only two in the lowest. These areas where disease burdens converge are not campaign-poor because they receive the most campaigns. Cluster-level visualisations are included in online supplemental figure 4.

### Campaign frequency and concurrency

A total of 1456 eligible campaigns were recorded across 43 countries in sub-Saharan Africa between 2019 and 2025, with annual volume rising from 167 in 2019 to 257 in 2025 (figure 4A). Across all country-years, 1216 pairs of campaigns targeting different intervention domains started within an 8-week window of one another in the same country, of which 599 (49%) were between two national-scope campaigns, 116 (10%) between two subnational campaigns and 501 (41%) of mixed scope. Of the 1216 pairs, 338 (28%) were highly compatible for integration, ie, those that started within two calendar months of one another, were occurring within 2 months, aligned in geographic scope, and with documented co-delivery precedence (online supplemental analysis S2; online supplemental table 4). Immunisation and NTD campaigns formed the most frequent domain pairing (373 pairs; mean compatibility score 0.57), followed by NTDs and nutrition (184 pairs; mean score 0.63) (figure 4B; online supplemental table 3).

**Figure 4:**
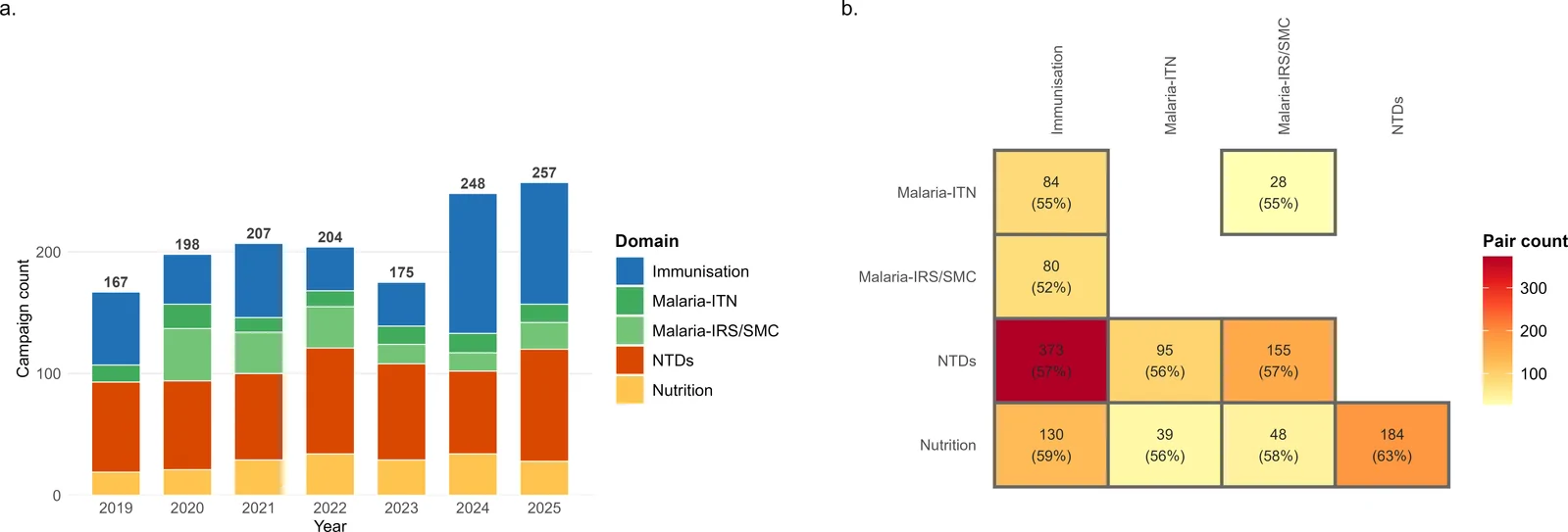
Campaign frequency and cross-domain concurrency. (A) Annual number of campaigns by intervention domain. (B) Proportion of country-years in which both domains of a pair had at least one active campaign. IRS, indoor-residual spraying; ITN, insecticide-treated net; NTD, neglected tropical disease; SMC, seasonal malaria chemoprevention.

### Missed integration opportunity

Temporal proximity narrows the pool of concurrent campaigns to those that could plausibly be integrated. Of the 1216 cross-domain pairs that started within an 8 week window in the same country, 338 pairs scored at or above 0.70 on the compatibility score yet were delivered as separate events. These separately delivered pairs were concentrated in the immunisation–neglected-tropical-disease and malaria–immunisation domain combinations, and in the countries carrying both the highest converging burden and the highest campaign frequency: Nigeria, Ethiopia and the Democratic Republic of the Congo, which together carried the largest numbers of separately delivered compatible pairs (figure 5; online supplemental table 4). The count of highly compatible pairs delivered separately was 338 under every temporal window between four and sixteen weeks, and lay between 237 and 338 across the alternative score thresholds and component weightings examined (online supplemental analysis S6; online supplemental tables 7–9).

**Figure 5:**
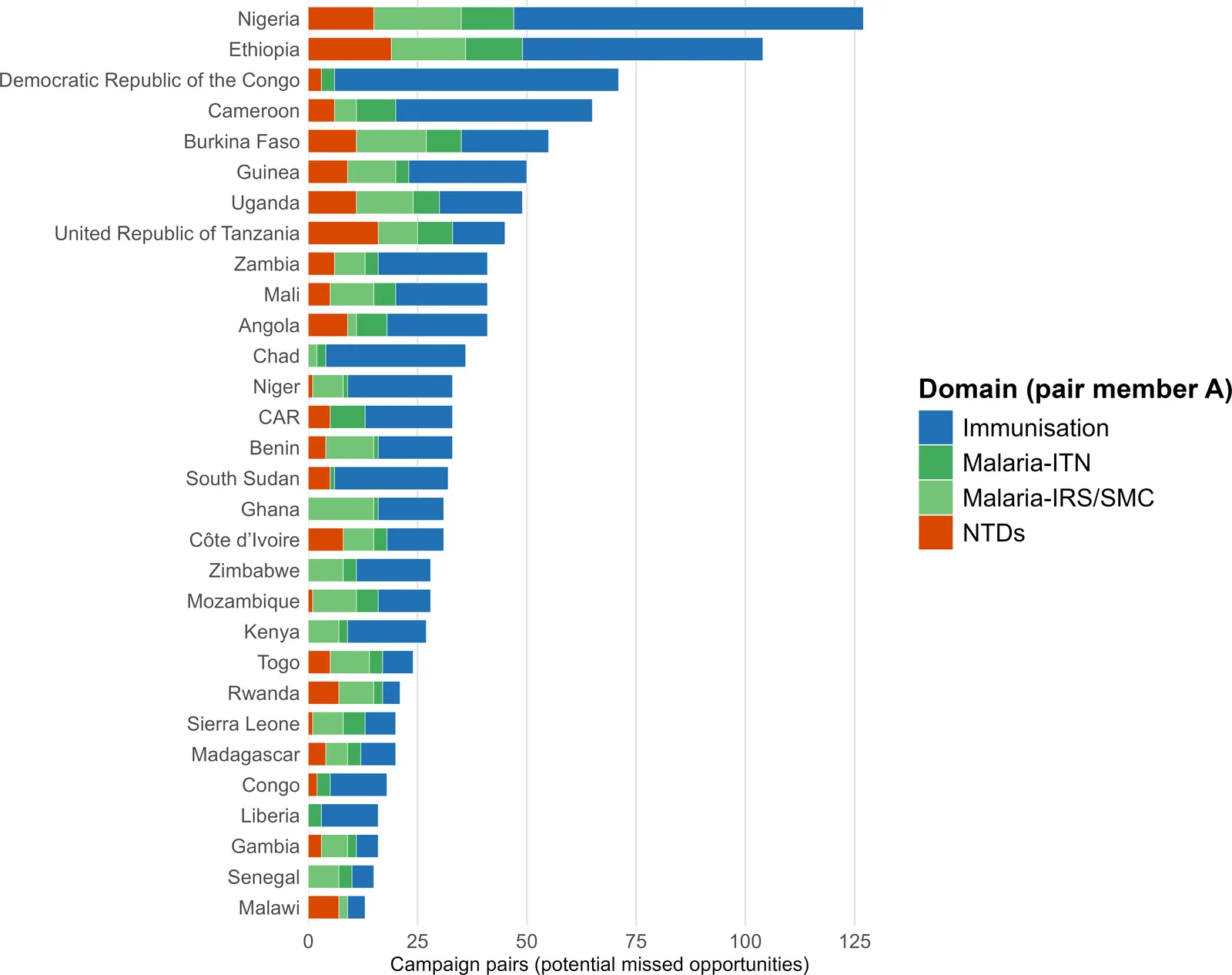
Countries ranked by number of separately delivered compatible pairs using compatibility score. IRS, indoor residual spraying; ITN, insecticide-treated net; ITN, insecticide-treated net; NTD, neglected tropical disease; SMC, seasonal malaria chemoprevention

## Discussion

Three findings emerge from analysing burden and campaign data at finer spatial resolution than either has previously been reported. First, malaria infection and childhood zero-dose prevalence co-occur within two-thirds of the countries examined, and their convergence concentrated in an identifiable set of Admin-1 units. Second, campaign frequency was highest in these same Admin-1 units. Third, compatible and temporally proximate campaigns are nonetheless delivered separately, and most often where disease burden is greatest. These results highlight that vertically organised programmes identify the same under-served populations but respond with parallel campaign delivery systems.

The campaign frequency is not in itself evidence of inefficiency since each campaign addresses a legitimate public-health objective, and concurrency reflects that the same children are eligible for several interventions in the same year. Vitamin A supplementation co-delivered with immunisation has decades of trial and operational support.^26 27^ Integrated delivery of neglected-tropical-disease mass drug administration with immunisation activities has been implemented in the United Republic of Tanzania and elsewhere with documented operational feasibility.^28 29^ Co-delivery of net distribution and seasonal malaria chemoprevention through community contact events has been piloted in West Africa.^30–32^ The co-occurrence reported here is consistent with earlier work at coarser resolution. The overlapping geography of the leading causes of death and disability in African children has been described at national and first subnational scale,^1^ zero-dose children have been mapped across 35 countries at subnational scale,^14^ and under-vaccination has been modelled geostatistically within individual fragile settings.^18^ The present estimates extend that evidence to the survey cluster across 18 countries and link it to the operational campaign calendar.

Evidence established here suggests that campaigns in the same country with similar seasonality and target populations are conducted as separate operations, each with its own microplan, logistics chain and community mobilisation procedure. Whether two concurrent campaigns can be integrated is a question that burden and scheduling data cannot answer on their own but require contextual assessment. Integration is constrained by factors that vary by setting and by intervention such as the safety and timing of co-administering products, including potential interactions between medicines and between medicines and vaccines; cold-chain and supply-chain capacity; the alignment of financing cycles and reporting requirements across donors and programmes; and the availability of health workers to deliver a combined package without displacing routine services.^13 15 33–37^ A campaign pair that is highly compatible on the scheduling criteria used here may be infeasible in a particular district for reasons the schedule does not capture. This compatibility score (figure 5) identifies candidate pairs but does not establish feasibility. The convergence map identifies where the assessment of integration would carry the most value. Seasonality further constrains what temporal proximity implies. Malaria campaigns, for example, are timed to the transmission season, so a shift of 1 month at the onset or in the middle of the wet season can materially change the effectiveness and the operational relevance of the intervention, even where the compatibility score is unchanged.

Two further implications emerge from these findings. First, the cluster-resolution convergence of malaria and zero-dose prevalence suggests that interventions targeting one burden encounter populations carrying the other. In these units, net-distribution and chemoprevention campaigns reach the same populations with under-vaccination. A campaign delivering one intervention is already in contact with the population carrying the other. Second, the coincidence of high burden, high campaign frequency and low integration suggests that the largest efficiency gains from integration, where it proves feasible, would arise in the highest-burden settings.^38^ However, these settings are where workforce and logistics constraints make feasibility hardest to achieve. This emphasises the need for further country-led feasibility assessments on integration alongside digital intervention that would support such delivery.^39^ The operational case for systematic integration has been set out separately, drawing on pilot evidence from Ethiopia, Nigeria and the Democratic Republic of the Congo.^17^ The present analysis provides the cluster-resolution empirical evidence underpinning that case across 18 countries.

There are several limitations to this study. Cluster-level survey proportions rely on small denominators rarely exceeding 25 children per cluster. The modelling exercise addressed this by borrowing strength across clusters, Admin-1 units and countries rather than by treating each observed proportion as an unadjusted estimate. Surveys were drawn from the most recent available round per country across 2015–2025, so the cross-country comparison spans a range of survey years. Two hundred and fifty Admin-1 units lacked cluster data for at least one indicator and could not be classified or included in the analysis. These gaps are non-random and concentrate in countries of sparse survey coverage. Estimates are reported at Admin-1 level because that is the level at which the DHS sampling frame is designed to be representative, whereas campaign microplanning in many countries is carried out at health-district level. The classification therefore identifies the regions in which assessment is required rather than the districts in which delivery is planned. The Admin-1 estimates are model expectations for a cluster of typical composition in each unit and do not incorporate survey sampling weights, so they are not population-weighted areal prevalences and should not be interpreted as such. The ITN access–use gap and campaign frequency enter the convergence classification as observed Admin-1 quantities rather than as modelled posteriors, so uncertainty is propagated for two of the four dimensions and not for the other two. The compatibility score treats temporal proximity symmetrically across the calendar, whereas the operational value of a 1 month shift depends on where it falls relative to the transmission season. No structured stakeholder engagement or country-level validation was undertaken as part of this analysis. The campaign scheduling records were compiled from country and partner reporting into the Campaign Hub, but that compilation is not equivalent to review of these estimates by the national programmes to which they refer. The convergence classification and the missed-opportunity counts therefore identify where the assessment of integration is required while the assessment itself requires the operational detail held by ministries of health and national programmes. This analysis, therefore, is intended to inform a country-led review of the Admin-1 classification, and of the campaign pairs identified within each country.

In conclusion, the spatial co-occurrence observed here could be used to transform health campaigns to a measurable advantage in coverage or cost when combined. This will require prospective contextual evaluations that are country-led and conducted as part of integration pilots across the various country contexts. The gaps in data due to the lack of available cluster-level require dense survey coverage or linkage to routine data in these same settings to improve evidence base.

## Supplementary material

10.1136/bmjgh-2026-025607 online supplemental file 1

## Data Availability

Data are available in a public, open access repository. Data are available upon reasonable request. All data relevant to the study are included in the article or uploaded as supplementary information.

## References

[1] Reiner RC, Hay SI, LBD Triple Burden Collaborators (2022). The overlapping burden of the three leading causes of disability and death in sub-Saharan African children. Nat Commun.

[2] Zheng J, Zhang D, Zhang S (2025). Global burden of malaria and neglected tropical diseases in children and adolescents, 1990-2019: a population-based, cross-sectional study. J R Soc Med.

[3] Banda GT, Deribe K, Davey G (2021). How can we better integrate the prevention, treatment, control and elimination of neglected tropical diseases with other health interventions? A systematic review. BMJ Glob Health.

[4] Krentel A, Rajabali A, Ogundahunsi O (2025). Opportunities and barriers arising from the COVID-19 pandemic for health campaign integration across immunizations, neglected tropical diseases, insecticide-treated bed nets, and vitamin A supplementation: A qualitative key informant interview study. *PLOS Glob Public Health*.

[5] World Health Organization (2025). World malaria report 2025: addressing inequity in the global malaria response.

[6] WHO/UNICEF (2025). Progress and challenges with achieving universal immunization coverage.

[7] Clark A, Sanderson C (2009). Timing of children’s vaccinations in 45 low-income and middle-income countries: an analysis of survey data. Lancet.

[8] Sayem ASM, Musuka G, Atuhebwe PL (2025). Childhood vaccination catch-up and recovery plans for mitigating immunity gap post the COVID-19 pandemic: A case study of selected African countries. Vaccine.

[9] World Health Organization (2024). Global report on neglected tropical diseases 2024.

[10] (2026). Alliance for malaria prevention. https://allianceformalariaprevention.com/.

[11] Health Campaign Effectiveness Coalition, Task Force for Global Health (2024). Collaborative action strategy for health campaign effectiveness 2024-2028.

[12] Boonstoppel L, Moi F, Banks C (2024). Cost of integrated immunization campaigns in Nigeria and Sierra Leone: bottom-up costing studies. BMC Health Serv Res.

[13] Gammino VM, Nuhu A, Gerber S (2014). An Evaluation of Polio Supplemental Immunization Activities in Kano, Katsina, and Zamfara States, Nigeria: Lessons in Progress. J Infect Dis.

[14] Kirkby K (2021). Zero-dose children: measuring access to vaccines and identifying missed opportunities for vaccination in 35 countries in sub-Saharan Africa. Bull World Health Organ.

[15] Wonodi C, Farrenkopf BA (2023). Zero-dose children in Africa: a scoping review of barriers and facilitators to immunisation. Vaccines (Basel).

[16] Oyo-Ita A (2023). Interventions for improving coverage of childhood immunisation in low- and middle-income countries. Cochrane Database Syst Rev.

[17] Impouma B, Mboussou F, Amani A (2026). From slogan to practice: integrating mass health campaigns in Africa. The Lancet.

[18] Alegana VA, Ticha JM, Mwenda JM (2024). Modelling the spatial variability and uncertainty for under-vaccination and zero-dose children in fragile settings. Sci Rep.

[19] Linksbridge SPC (2025). Campaign hub: global health campaign calendar and co-delivery database.

[20] United Nations DoE, Social Affairs PD (2024). World population prospects 2024.

[21] Riebler A, Sørbye SH, Simpson D (2016). An intuitive Bayesian spatial model for disease mapping that accounts for scaling. Stat Methods Med Res.

[22] Simpson D, Rue H, Riebler A (2017). Penalising Model Component Complexity: A Principled, Practical Approach to Constructing Priors. Statist Sci.

[23] Besag J, York J, Mollie A (1991). Bayesian image restoration, with two applications in spatial statistics. Ann Inst Stat Math.

[24] Rue H, Martino S, Chopin N (2009). Approximate Bayesian Inference for Latent Gaussian models by using Integrated Nested Laplace Approximations. Journal of the Royal Statistical Society Series B.

[25] CAMBER (2020). Campaign effectiveness landscape and case for action.

[26] Imdad A, Mayo-Wilson E, Herzer K (2017). Vitamin A supplementation for preventing morbidity and mortality in children from six months to five years of age. Cochrane Database Syst Rev.

[27] Wondie WT, Zemariam AB, Gedefaw GD (2025). Vitamin A supplementation coverage and its associated factors among children 6-59 months of age in Ethiopia: a systematic review and meta-analysis. Front Public Health.

[28] Hotez PJ, Molyneux DH, Fenwick A (2006). Incorporating a rapid-impact package for neglected tropical diseases with programs for HIV/AIDS, tuberculosis, and malaria. PLoS Med.

[29] Mwingira UJ (2016). Integrating neglected tropical disease and immunization programs: experiences of the Tanzanian Ministry of Health. Am J Trop Med Hyg.

[30] Djedanem M, Salé NM, Aminou EYYM (2025). Status and prospects of seasonal malaria chemoprevention among children in Sahelian countries: A systematic review and meta-analysis. *PLOS Glob Public Health*.

[31] Leslie J (2013). Neglected tropical diseases: comparison of costs of integrated and vertical preventive chemotherapy in Niger. Int Health.

[32] Fuller BB (2023). Contextual determinants of mass drug administration performance: modelling fourteen years of lymphatic filariasis treatments in West Africa. PLoS Negl Trop Dis.

[33] Wariri O (2024). Multi-level determinants of timely routine childhood vaccinations in The Gambia: findings from a nationwide analysis. Vaccine.

[34] Ashok A, Brison M, LeTallec Y (2017). Improving cold chain systems: challenges and solutions. Vaccine.

[35] Wonodi C, Farrenkopf BA (2023). Defining the zero-dose child: a comparative analysis of two approaches across 82 low- and middle-income countries. Vaccines (Basel).

[36] Chouaid A (2025). Community engagement in mass drug administration participatory interventions: a scoping review. PLoS Negl Trop Dis.

[37] Shah MP, Morgan CJ, Beeson JG (2024). Integrated Approaches for the Delivery of Maternal and Child Health Services with Childhood Immunization Programs in Low- and Middle-Income Countries: Systematic Review Update 2011-2020. Vaccines (Basel).

[38] Wilder-Smith A (2017). The public health value of vaccines beyond efficacy: methods, measures and outcomes. BMC Med.

[39] Bhattacharya A (2025). Systematic review of mHealth and digital health interventions to improve childhood vaccination uptake in 19 Sub-Saharan African countries. PLoS One.

