# Supplementary material for: Cluster-level convergence of malaria infection, childhood zero-dose prevalence and health-campaign frequency in sub-Saharan Africa: a Bayesian analysis of 18 countries
Source: BMJ Glob Health. 2026 Nov 11;11(11):e025607. doi: 10.1136/bmjgh-2026-025607 (PMC13756409; doi:10.1136/bmjgh-2026-025607)
Supplement: Supplementary file 1 [file bmjgh-11-11-s001.pdf]

Cluster-level convergence of malaria infection, childhood zero-dose prevalence and health-campaign frequency in sub-Saharan Africa: a Bayesian analysis of 18 countries

Victor Alegana[1*], Bridget Farham[1], Franck Mboussou[2*], Dorothy Achu[1], Benido Impouma[1]

Supplemental Information

This document reports the sub-national (Admin-1) and national analyses that support the cluster-level results in the main text, together with Supplemental tables, figures and model specifications.

Supplemental Table 1a: Surveys contributing to the cluster-level analysis.

Most recent Demographic and Health Survey or Malaria Indicator Survey per country, with the number of paired survey clusters contributing to the cluster-level model and the posterior correlation between malaria rapid diagnostic test positivity and zero-dose prevalence. Clusters with fewer than ten tested children (M1) or fewer than ten vaccination-eligible children (M2) were excluded before fitting.

Country	ISO3	Survey	Survey year	Paired clusters	Correlation, posterior mean	95% credible interval
Angola	AGO	AO2023DHS	2023	481	0.31	0.23 to 0.40
Benin	BEN	BJ2017DHS	2017	481	0.41	0.32 to 0.49
Burkina Faso	BFA	BF2021DHS	2021	462	-0.04	-0.13 to 0.05
Burundi	BDI	BU2016DHS	2016	518	0.01	-0.07 to 0.10

Cameroon	CMR	CM2018DHS	2018	341	0.27	0.16 to 0.37
Côte d'Ivoire	CIV	CI2021DHS	2021	422	0.20	0.11 to 0.30
Democratic Republic of the Congo	COD	CD2023DHS	2023	738	0.32	0.25 to 0.38
Gabon	GAB	GA2019DHS	2019	323	0.30	0.20 to 0.41
Ghana	GHA	GH2022DHS	2022	389	0.14	0.04 to 0.24
Madagascar	MDG	MD2021DHS	2021	501	0.34	0.26 to 0.42
Malawi	MWI	MW2024DHS	2024	431	-0.05	-0.14 to 0.05
Mali	MLI	ML2023DHS	2023	331	-0.03	-0.14 to 0.08
Mozambique	MOZ	MZ2022DHS	2022	345	0.43	0.33 to 0.52
Nigeria	NGA	NG2024DHS	2024	1015	0.38	0.32 to 0.43
Rwanda	RWA	RW2019DHS	2019	377	0.03	-0.07 to 0.13
Senegal	SEN	SN2023DHS	2023	385	0.39	0.30 to 0.49
Uganda	UGA	UG2016DHS	2016	423	0.02	-0.08 to 0.11
United Republic of Tanzania	TZA	TZ2022DHS	2022	433	0.16	0.07 to 0.25
**Total (18 countries)**				**8396**		

Analysis S1. Cluster-level three-level BYM2 co-burden analysis

Model Specification

Survey cluster data provided the unit of observation. Two indicators were modelled: M1, RDT positivity in children aged 6–59 months, and M2, zero-dose prevalence in children aged 12–23 months. Clusters with fewer than ten sampled children ($n_i^k < 10$) were excluded before model fitting.

A three-level hierarchical binomial model was specified. For cluster $i = 1, \dots, N$ and indicator k:

$$Y_i^k \mid \pi_i^k \sim Binomial(n_i^k, \pi_i^k)$$

$$logit(\pi_i^k) = \alpha^k + u^k{}_{t(i)} + s^k{}_{h(i)} + v_i^k$$

Three random effects partition variance across nested scales. The term $v_i^k \sim \mathcal{N}(0, \sigma v^2)$ captured the cluster-level IID overdispersion — between-cluster heterogeneity within Admin-1 units not attributable to the spatial field. The term $s_j^k \sim BYM2(\varphi^k, \sigma s^2)$ is the Admin-1 spatially structured and unstructured random effect following [1]. The term $u_c^k \sim \mathcal{N}(0, \sigma u^2)$ is a country-level IID baseline capturing between-country differences not explained by the spatial field.

Throughout, i indexes the survey cluster, j the Admin-1 unit and c the country; the superscript k denotes the indicator. The two mapping functions carry a cluster to the areal units containing it: $h(i)$ returns the Admin-1 unit and $t(i)$ the country in which cluster i falls. In the likelihood, n_i^k is

the number of children tested for M1 (Malaria prevalence), or eligible for vaccination for M2 (Zero-dose prevalence), in cluster i, and $Y_{\mathrm{i}}^{\mathrm{k}}$ the number positive or zero-dose. The three variance components are σv^2, the marginal variance of the cluster-level IID term; σs^2, the marginal variance of the Admin-1 BYM2 term; and σu^2, the marginal variance of the country-level IID term, with τv, τs and τu the corresponding precisions. φ^{k} is the BYM2 mixing parameter, the proportion of Admin-1 variance that is spatially structured.

Prior distributions

PC priors [2] were assigned to the precision hyperparameters of all three random effects, anchored at the respective null models of no random variation. The PC prior on precision encodes that the marginal standard deviation on the log-odds scale exceeds 1 with probability 0.01. This placed a small prior mass on an implausibly large variance.

$$P(\varphi^{\mathrm{k}} < 0.5) = 2/3$$

$$P(1/\sqrt{\tau s^{\mathrm{k}}} > 1) = 0.01 \quad P(1/\sqrt{\tau u^{\mathrm{k}}} > 1) = 0.01 \quad P(1/\sqrt{\tau v^{\mathrm{k}}} > 1) = 0.01$$

Admin-1 posterior estimates

Admin-1 posterior means were derived without a separate model fit by appending one prediction row per Admin-1 unit to the observed cluster stack, with the outcome set to NA. Each prediction row received a unique cluster index exceeding the maximum observed cluster index, ensuring the cluster IID random effect contributed zero to the predicted value at that row. The Admin-1 fitted value reflected:

$$logit(\hat{\pi}_j^k) = \hat{\alpha}^{\mathrm{k}} + \hat{\mathrm{u}}_{t(j)}^k + \acute{\mathrm{s}}_j^k$$

where the cluster overdispersion term is absent. This was the appropriate population-level areal estimate.

Co-burden classification methodology

Admin-1 posterior means from M1 and M2 were each assigned a tertile rank T_j^{M1} and $T_j^{M2} \in [1, 2, 3]$. Posterior mean estimates $\hat{\pi}_j^{(k)}$ were extracted for each indicator. Each unit was assigned a tertile score $T_j^{(k)} \in [1,2,3]$ based on its rank within the posterior mean distribution across all units:

$$T_j^{(k)} = \begin{cases} 1 & \hat{\pi}_j^{(k)} \le Q_{33}\left(\hat{\boldsymbol{\pi}}^{(k)}\right) \\ 2 & Q_{33} < \hat{\pi}_j^{(k)} \le Q_{67} \\ 3 & \hat{\pi}_j^{(k)} > Q_{67}\left(\hat{\boldsymbol{\pi}}^{(k)}\right) \end{cases}$$

A composite convergence score was computed by summing tertile ranks across the three indicators:

$$S_j = T_j^{\text{rdt}} + T_j^{\text{zdose}} + T_j^{\text{gap}}, \quad S_j \in [3, \dots, 9]$$

where T_j^{rdt}, T_j^{zdose} and T_j^{gap} are the tertile ranks, taking the value 1, 2 or 3, of respectively the posterior mean malaria RDT positivity, the posterior mean zero-dose prevalence, and the ITN access–use gap in Admin-1 unit j. The ITN access–use gap is the difference, in percentage points, between the survey-weighted proportion of households owning at least one ITN and the survey-weighted proportion of children under five who slept under an ITN the previous night. S_j therefore ranges from 3, where the unit is in the lowest tertile on all three indicators, to 9, where it is in the highest tertile on all three.

A four-dimensional score incorporating campaign frequency was also computed:

$$S_j^{(4)} = S_j + T_j^{\text{camp}}, \quad S_j^{(4)} \in [4, \dots, 12]$$

where T_j^{camp} is the tertile rank of the count of AFRO campaigns conducted in unit j during 2019–2025. Units were classified into burden convergence classes:

Score (S_j)	Class
8–9	Triple burden
5–7	Dual burden
3–4	Low burden

For the four-dimensional score:

Score ($S_j^{(4)}$)	Class
9–12	Triple-plus burden
7–8	Triple burden
5–6	Dual burden

3–4	Low burden

Units where posterior estimates were unavailable for either indicator were excluded from classification and rendered grey in choropleth maps.

Supplemental Table 1b: Per-country posterior Bayesian correlation between cluster-level malaria RDT positivity and zero-dose prevalence. Standardised regression slope equals the Pearson correlation. Ordered by posterior mean.

Country	Paired clusters	Posterior mean ρ	95% BCI	Classification
Mozambique	345	0.427	0.331–0.523	Positive (BCI > 0)
Benin	481	0.407	0.325–0.489	Positive (BCI > 0)
Senegal	385	0.395	0.303–0.487	Positive (BCI > 0)
Nigeria	1015	0.376	0.319–0.433	Positive (BCI > 0)
Madagascar	501	0.338	0.256–0.421	Positive (BCI > 0)
Democratic Republic of the Congo	738	0.315	0.247–0.384	Positive (BCI > 0)
Angola	481	0.313	0.228–0.398	Positive (BCI > 0)
Gabon	323	0.304	0.200–0.408	Positive (BCI > 0)
Cameroon	341	0.265	0.162–0.368	Positive (BCI > 0)
Côte d'Ivoire	422	0.203	0.109–0.297	Positive (BCI > 0)
United Republic of Tanzania	433	0.159	0.066–0.252	Positive (BCI > 0)
Ghana	389	0.141	0.042–0.240	Positive (BCI > 0)
Rwanda	377	0.030	-0.071–0.131	Uncertain
Uganda	423	0.018	-0.077–0.114	Uncertain
Burundi	518	0.015	-0.071–0.101	Uncertain
Mali	331	-0.033	-0.141–0.075	Uncertain
Burkina Faso	462	-0.043	-0.134–0.048	Uncertain
Malawi	431	-0.046	-0.140–0.049	Uncertain

Model validation

Predictive performance was evaluated via a 20% random holdout per indicator (set.seed(2026L)). Holdout cluster rows were appended to the training stack with $y\_int = NA$, using the same prediction-row mechanism as the Admin-1 estimation. Model structure, formula,

prior distributions, and spatial graph were identical to the main analysis; no parameters were altered. Root mean squared error (RMSE) was computed as:

$$RMSE = \sqrt{[mean((y_i/n_i - \hat{\pi}_i)^2)]}$$

where y_i/n_i is the observed cluster proportion and $\hat{\pi}_i$ is the posterior mean from the holdout model. Calibration was assessed via a scatter plot of observed against predicted proportions, with the $y = x$ identity line as the reference. Separate holdout models were fitted for M1 and M2.

Supplemental Table 2 : Posterior summaries of the three-level cluster BYM2 hyperparameters for RDT positivity (M1) and zero-dose prevalence (M2). Phi for id is the Admin-1 BYM2 mixing parameter ϕ.

Indicator	Hyperparameter	Posterior mean	SD	95% BCI
RDT positivity	Precision for country_idx	1.131	0.504	0.432–2.375
RDT positivity	Precision for id	0.259	0.031	0.203–0.324
RDT positivity	Phi for id	0.998	0.001	0.996–1.000
RDT positivity	Precision for cluster_idx	0.710	0.026	0.660–0.762
Zero-dose	Precision for country_idx	0.718	0.212	0.398–1.226
Zero-dose	Precision for id	1.311	0.203	0.960–1.758
Zero-dose	Phi for id	0.789	0.082	0.596–0.913
Zero-dose	Precision for cluster_idx	1.494	0.039	1.419–1.573

Analysis S2 (a). Missed co-delivery opportunities

Campaign pairs sharing a country and calendar year were scored for co-delivery compatibility using a three-component continuous score (adapted from the IA2030 DSI Working Group, following the shared-planning and calendar-alignment strategy set out in the campaign effectiveness landscape analysis prepared for the Task Force for Global Health, which identified the absence of a framework for assessing coordination opportunities across campaign domains as a priority data gap): temporal proximity (1.0 if start months were identical, 0.5 if one calendar month apart, 0 if two calendar months apart); geographic-scope alignment (1.0 for two national campaigns, 0.7 for two subnational campaigns, 0.5 for mixed scope); and documented co-delivery

precedence (0.8 if either campaign had a co-delivery record, 0.5 otherwise), weighted 0.40, 0.30, and 0.30 respectively. Campaigns were assigned one primary intervention domain in the following priority order: Malaria (ITN), Malaria (IRS/SMC), Nutrition, Neglected Tropical Diseases, and Immunisation; Polio (oral and inactivated polio vaccine campaigns) was classified within the Immunisation domain in the primary analysis, reflecting the shared Expanded Programme on Immunization (EPI) operational structure. A sensitivity analysis with Polio as a distinct domain is presented separately. Outbreak-response campaigns were excluded except for yellow fever and measles events (562 records removed), leaving 1,667 campaigns eligible. Only pairs targeting different primary domains and scoring at least 0.70 within a two-calendar-month window were classified as high-compatibility co-delivery opportunities. Across the WHO African Region 2019–2025, 3,614 cross-domain campaign pairs were identified in the same country-year, of which 1,216 (34%) occurred within an eight-week window; 338 (28%) of those scored at or above 0.70 and were delivered separately. Per-country counts are reported in Table S4.

Supplemental Table 3: Cross-domain campaign pairs in the WHO African Region, 2019–2025, by intervention domain pair (five-domain main analysis). Ordered by total pair count. High-compat. = pairs with score ≥0.70.

Domain pair	Total pairs	Mean compat. score	High-compat. pairs	High-compat. (%)
Immunisation × NTDs	373	0.57	111	29.8
NTDs × Nutrition	184	0.63	72	39.1
Malaria-IRS/SMC × NTDs	155	0.57	27	17.4
Immunisation × Nutrition	130	0.59	42	32.3
Malaria-ITN × NTDs	95	0.56	24	25.3
Immunisation × Malaria-ITN	84	0.55	22	26.2
Immunisation × Malaria-IRS/SMC	80	0.52	15	18.8
Malaria-IRS/SMC × Nutrition	48	0.58	10	20.8
Malaria-ITN × Nutrition	39	0.56	10	25.6
Malaria-IRS/SMC × Malaria-ITN	28	0.55	5	17.9

Supplemental Table 4: National counts of separately delivered high-compatibility campaign pairs (compatibility score ≥0.70, within 2 calendar months, different primary domain) and mean compatibility score

Country	High-compatibility pairs	Mean compatibility score
Ethiopia	33	0.752
Nigeria	33	0.794
Democratic Republic of the Congo	28	0.748
Angola	17	0.775
Burkina Faso	17	0.815
Cameroon	16	0.783
Uganda	16	0.844
Central African Republic	15	0.781
Guinea	13	0.834

Mali	13	0.808
United Republic of Tanzania	11	0.755
Chad	10	0.775
Madagascar	8	0.831
Benin	7	0.756
Burundi	7	0.843
Liberia	7	0.829
Niger	7	0.764
Zambia	7	0.819
Zimbabwe	7	0.764
Cote d'Ivoire	6	0.818
Gambia	6	0.77
Mozambique	6	0.722
South Sudan	6	0.797
Ghana	5	0.76
Kenya	5	0.794
Malawi	5	0.78
Congo	4	0.872
Gabon	3	0.83
Sierra Leone	3	0.843
Togo	3	0.813
Comoros	2	0.895
Guinea-Bissau	2	0.85
Mauritania	2	0.85
Rwanda	2	0.895
Senegal	2	0.82
Botswana	1	0.85
Eswatini	1	0.94
Namibia	1	0.74
South Africa	1	0.85

Analysis S2(b). Sensitivity analysis: Polio (OPV/IPV) as a separate intervention domain

To assess whether classifying Polio campaigns within the Immunisation domain materially affects estimates of missed integration opportunities, the analysis was repeated using six primary domains, with Polio treated as distinct from general Immunisation, reflecting the separate scheduling and logistics pathways of national immunisation days (NIDs) and sub-national immunisation days (SNIDs). Filtering, scoring, and the high-compatibility threshold were identical to the main analysis. Under the six-domain scheme, 1,312 feasible cross-domain pairs were identified within an eight-week window, of which 357 (27%) were high-compatibility missed co-delivery opportunities. The NTDs × Polio pair was the most frequent (237 pairs, mean compatibility score 0.58); the Immunisation × Polio pair contributed 96 pairs (mean score 0.54). Annual campaign volume totals are identical to the main analysis — the same 1,667 campaigns are classified under a six-domain scheme.

Analysis S3. DTP1-to-DTP3 dropout spatial variability

The DTP1-to-DTP3 dropout rate (the proportion of children receiving DTP1 but not completing DTP3) was modelled as a binomial outcome with the DTP1 count as the effective denominator, using the same Admin-1 BYM2 structure and priors:

$$\mathrm{DR}_i = \frac{\hat{p}_i^{\mathrm{dtp1}} - \hat{p}_i^{\mathrm{dtp3}}}{\hat{p}_i^{\mathrm{dtp1}}}, \quad \hat{p} > 0$$

where $\hat{p}_i^{\mathrm{dtp1}}$ and $\hat{p}_i^{\mathrm{dtp3}}$ are the estimated DTP1 and DTP3 coverage proportions in ADMIN-1 unit i.

Within-country spatial inequity was summarised by the coefficient of variation (CV) of posterior mean dropout across a country's Admin-1 units. The widest within-country variation occurred in Mali (CV 0.49), Ethiopia (0.46), Kenya (0.42) and Mozambique (0.41); the highest mean dropout occurred in the Democratic Republic of the Congo (0.49), Angola and Guinea (0.44 each) (Supplemental Table 5; Supplemental Fig. S1).

Analysis S4. Association between ITN gap, zero-dose and dropout

A Gaussian BYM2 regression related the Admin-1 ITN access–use gap and DTP dropout to zero-dose prevalence, adjusting for spatially structured confounding, with mean-centred predictors and the BYM2 priors. Across 343 Admin-1 units (excluding Lesotho), zero-dose prevalence and DTP dropout were strongly positively associated (empirical Pearson correlation 0.82), indicating that areas of high under-vaccination also retain children who start but do not complete the schedule

Supplemental Table 5: Within-country DTP1-to-DTP3 dropout: mean posterior dropout, coefficient of variation across Admin-1 units, and range in percentage points (Analysis S3). Ordered by CV.

Country	Admin-1 units	Mean dropout	CV of dropout	Range (pp)
Mali	9	0.288	0.492	38.9
Ethiopia	11	0.273	0.462	42.6
Kenya	47	0.148	0.415	30.1
Mozambique	11	0.296	0.414	35.6
Madagascar	23	0.207	0.373	26.0
Burkina Faso	13	0.149	0.366	21.1
Democratic Republic of the Congo	26	0.486	0.315	56.6

Benin	12	0.208	0.301	18.8
Cameroon	12	0.182	0.275	15.9
United Republic of Tanzania	9	0.127	0.271	9.9
Guinea	8	0.443	0.253	32.3
Angola	18	0.443	0.245	37.1
Burundi	18	0.087	0.237	7.5
Côte d'Ivoire	14	0.267	0.219	17.8
Gabon	11	0.238	0.216	18.2
Nigeria	6	0.219	0.211	9.8
Zimbabwe	10	0.138	0.207	7.3
Senegal	14	0.144	0.198	10.9
Ghana	16	0.146	0.197	9.7
Uganda	15	0.218	0.188	15.9
Cameroon	4	0.295	0.183	12.8
Zambia	10	0.136	0.179	6.6
Liberia	6	0.331	0.149	11.7
Malawi	3	0.191	0.144	5.4
Sierra Leone	5	0.261	0.142	7.3
Rwanda	5	0.063	0.088	1.6

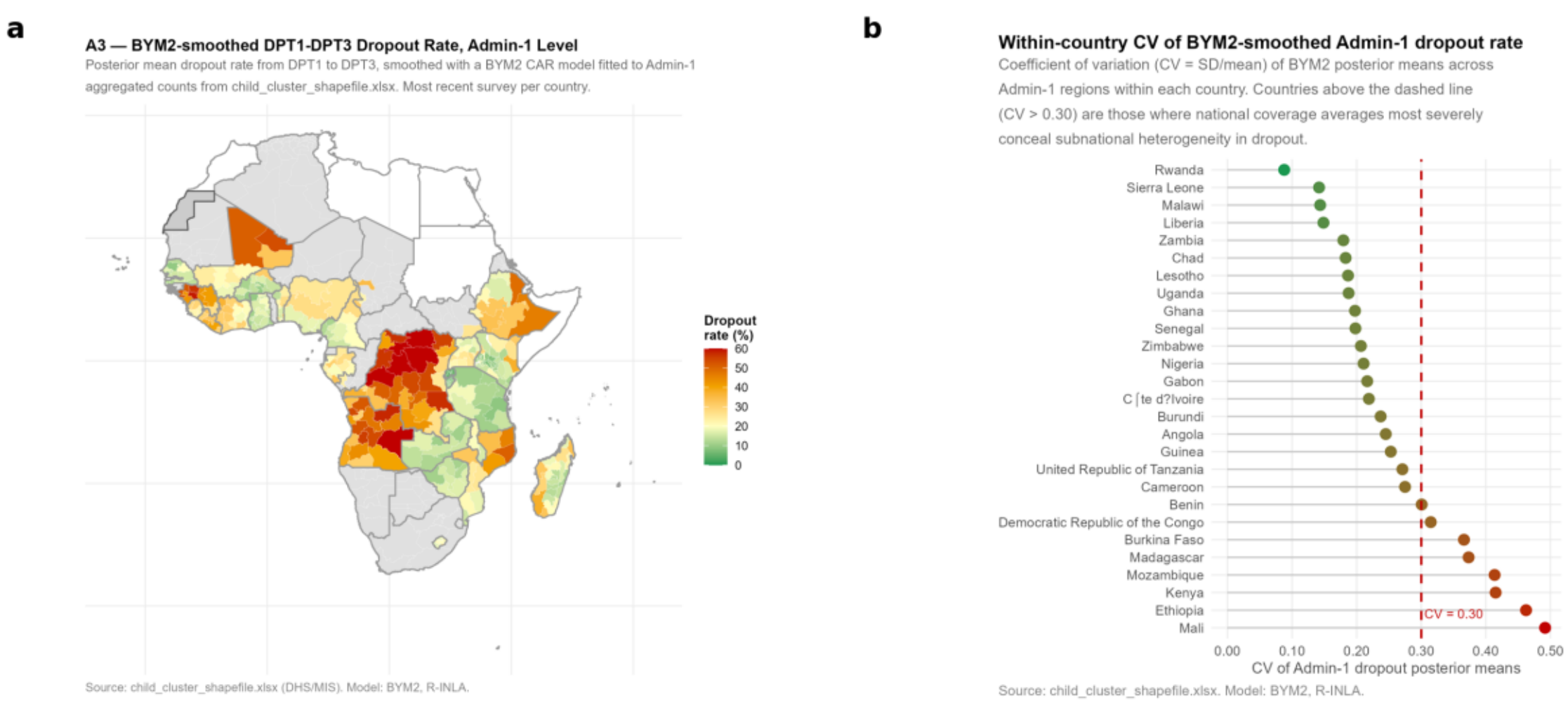

Supplemental Figure 1: DTP1-to-DTP3 dropout (Analysis S3). a, Posterior mean Admin-1 dropout rate. b, Within-country coefficient of variation of dropout, by country.

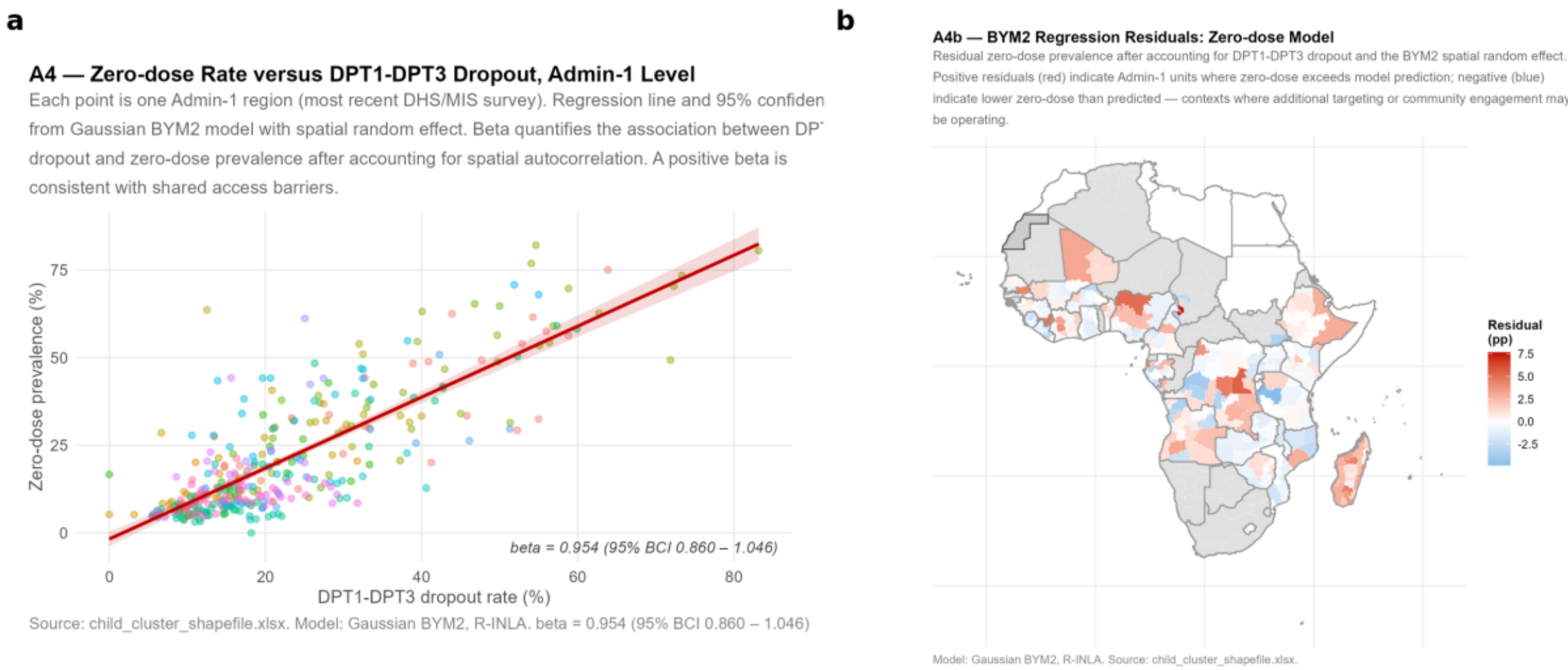

Supplemental Figure 2: Zero-dose and DPT1-DPT3 dropout rate association (Analysis S4). a, Admin-1 zero-dose prevalence against DTP dropout with the BYM2 regression fit. b, Spatial distribution of regression residuals.

Prior sensitivity

Penalised-complexity priors were anchored at the null model of no random variation. A sensitivity comparison against weakly informative Gamma priors on the precisions (shape 1, rate 5×10^{-5}) and uniform priors on the BYM2 mixing parameter indicated that posterior mean estimates were robust to prior specification across the BYM2 models.

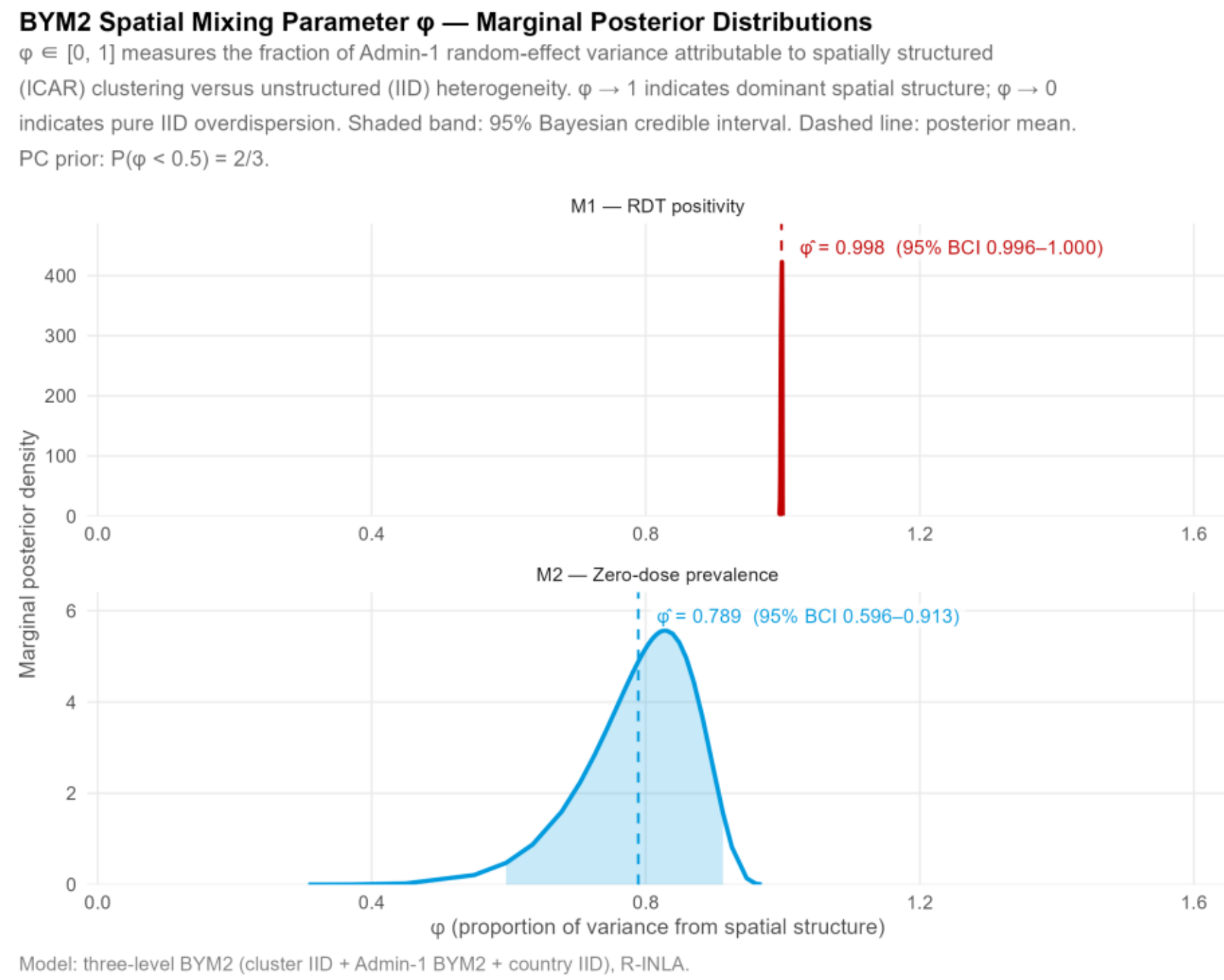

Supplemental Figure 3: Posterior density of the BYM2 mixing parameter ϕ. Posterior distributions of ϕ for the RDT-positivity and zero-dose models, indicating the proportion of Admin-1 variance attributable to spatial structure.

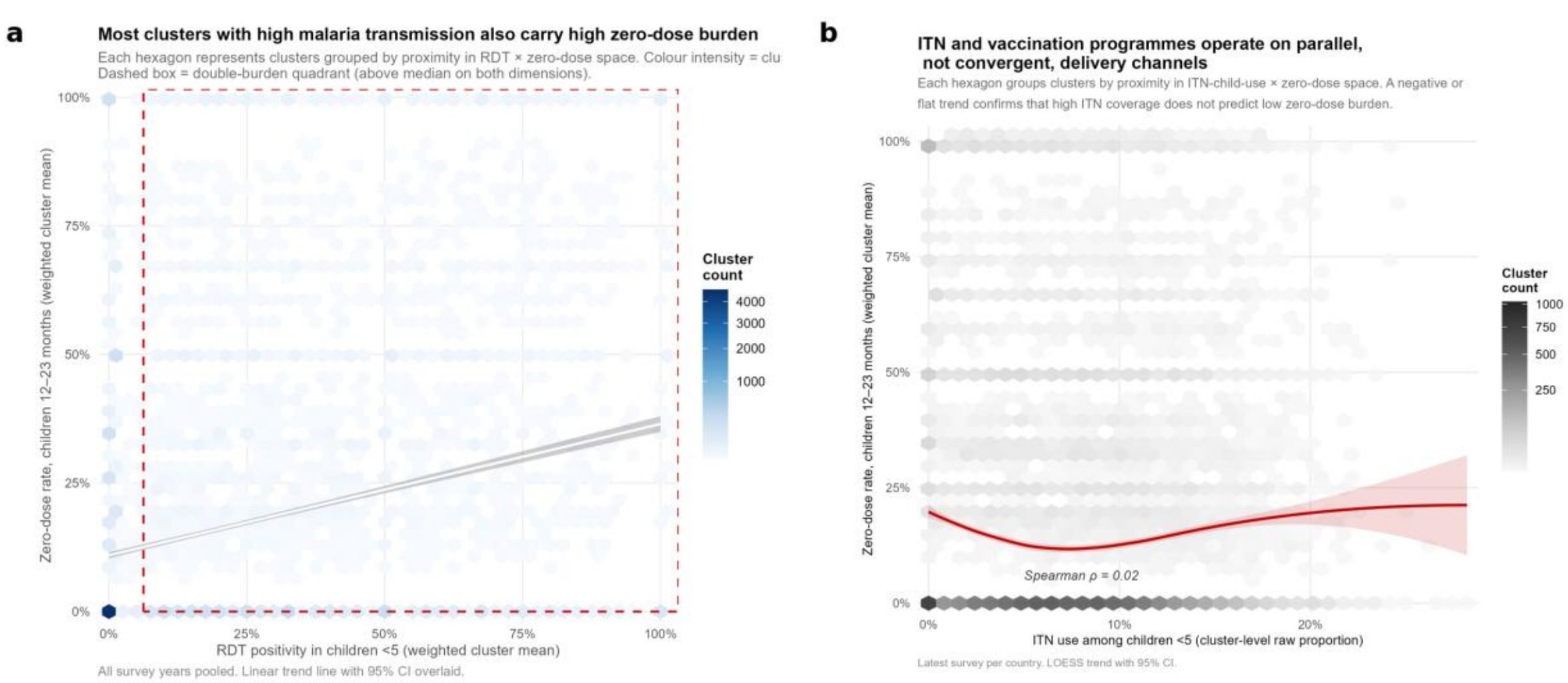

Supplemental Figure 4: Cluster-level supporting visualisations. a, Hexbin density of cluster-level malaria RDT positivity against zero-dose prevalence. b, Cluster-level ITN and vaccination co-delivery relationship.

Analysis S5. Per-country cluster-level correlation

The association between the two modelled indicators was estimated at the cluster rather than at the Admin-1 level, because the survey cluster is the unit at which both indicators are observed on the same population. For each country with at least 20 paired clusters, cluster-level RDT positivity and zero-dose prevalence were standardised to zero mean and unit variance, that is z-scored, and related by a Gaussian regression in R-INLA, so that the posterior slope is the Bayesian equivalent of the Pearson correlation coefficient. Because the two indicators are fitted independently in Analysis S1 rather than through a joint model with a shared latent field, the correlation reported here is estimated from the data rather than induced by the model specification. Posterior means and 95% credible intervals for all 18 countries, together with the number of paired clusters contributing to each, are given in Supplemental Table 1a and Supplemental Table 1b.

Analysis S6. Sensitivity of the campaign compatibility score

The compatibility score combines temporal proximity, geographic-scope alignment and documented co-delivery precedence, weighted 0.40, 0.30 and 0.30 respectively, with pairs scoring at least 0.70 within an eight-week window classified as high-compatibility co-delivery opportunities. No dataset links the features of a campaign pair to the realised success of co-delivery, so the weights are a stated operational prior rather than an estimated quantity. The count of high-compatibility pairs was therefore recomputed across the attainable values of the score, alternative temporal windows, alternative thresholds and alternative component weightings. The analysis set is the 1,456 eligible campaigns recorded in the Campaign Hub for the WHO African Region between 2019 and 2025, from which 1,216 cross-domain pairs fell within the eight-week window.

Each component of the score is discrete, so the score itself takes only 15 distinct values (Supplemental Table 6). The threshold of 0.70 is one of them, corresponding exactly to the weakest same-month configuration: two campaigns starting in the same calendar month, mixed in geographic scope, and with no documented co-delivery precedent. Every pair admitted at 0.70 therefore either starts in the same calendar month, or starts one month apart and is national in scope for both campaigns with a documented co-delivery record, which scores 0.74.

The temporal component of the score falls to zero at a gap of two calendar months, so the highest score attainable by a pair separated by two or more months is 0.54. No pair separated by more

than one month can reach 0.70 under any of the weightings examined. Widening the eligibility window from four to sixteen weeks therefore leaves the identified set unchanged at 338 pairs across 39 countries and changes only the denominator, from 765 pairs to 1,923 (Supplemental Table 7). The eight-week window fixes the denominator against which high-compatibility pairs are expressed rather than the set of pairs identified.

Varying the threshold within the eight-week window gives 535 pairs (44.0%) at 0.65, 338 (27.8%) at 0.70, 192 (15.8%) at 0.75 and 138 (11.3%) at 0.80 (Supplemental Table 8). The count scales with the threshold, but the direction of the findings is unchanged across the range examined.

Varying the component weights at the 0.70 threshold gives 254 pairs (20.9%) under equal weighting, 338 (27.8%) with temporal proximity weighted at 0.50, 254 (20.9%) with temporal proximity discounted to 0.30, and 237 (19.5%) with documented precedence weighted at 0.50 (Supplemental Table 9). Across every specification examined the count of highly compatible cross-domain pairs delivered as separate operations lies between 237 and 338, that is between a fifth and a little over a quarter of temporally proximate cross-domain pairs (Supplemental Figure 5).

Supplemental Table 6: Attainable values of the campaign compatibility score, eight-week window (Analysis S6). Each of the three components is discrete, so the score takes 15 distinct values. The main-analysis threshold of 0.70 is itself an attainable value, corresponding to two campaigns starting in the same calendar month with mixed geographic scope and no documented co-delivery precedent.

Score	Pairs at this score	Cumulative pairs at or above	% of the 1,216 pairs in window
**0.94**	37	37	3.0
**0.85**	101	138	11.3
**0.79**	37	175	14.4
**0.76**	17	192	15.8
**0.74**	62	254	20.9
**0.70**	84	338	27.8
**0.65**	197	535	44.0
**0.59**	50	585	48.1
**0.56**	32	617	50.7
**0.54**	68	685	56.3
**0.50**	148	833	68.5
**0.45**	168	1001	82.3
**0.39**	49	1050	86.3
**0.36**	33	1083	89.1
**0.30**	133	1216	100.0

Supplemental Table 7: Sensitivity of the high-compatibility count to the temporal window, with the scoring function held fixed (Analysis S6). Because the temporal component is zero at a gap

of two calendar months, no pair separated by more than one month can reach 0.70; widening the window changes only the denominator.

Window	Calendar-month gap	Cross-domain pairs in window	High-compatibility pairs (≥0.70)	% of pairs in window	Countries
**4 weeks**	≤ 1	765	338	44.2	39
**8 weeks (main)**	≤ 2	1,216	338	27.8	39
**12 weeks**	≤ 3	1,587	338	21.3	39
**16 weeks**	≤ 4	1,923	338	17.6	39

Supplemental Table 8: Sensitivity of the high-compatibility count to the score threshold, eight-week window (Analysis S6). Thresholds falling between attainable score values return identical counts.

Threshold	High-compatibility pairs	% of the 1,216 pairs in window
**0.60**	535	44.0
**0.65**	535	44.0
**0.70 (main)**	338	27.8
**0.75**	192	15.8
**0.80**	138	11.3

Supplemental Table 9: Sensitivity of the high-compatibility count to the component weighting, eight-week window and a threshold of 0.70 (Analysis S6). Weights are given in the order temporal proximity, geographic-scope alignment, documented co-delivery precedence.

Weighting	High-compatibility pairs	% of the 1,216 pairs in window	Mean score
**0.40 / 0.30 / 0.30 (main)**	338	27.8	0.576
**0.34 / 0.33 / 0.33 (equal)**	254	20.9	0.592
**0.50 / 0.25 / 0.25 (timing-weighted)**	338	27.8	0.551
**0.30 / 0.35 / 0.35 (timing-discounted)**	254	20.9	0.600
**0.30 / 0.20 / 0.50 (precedence-weighted)**	237	19.5	0.573

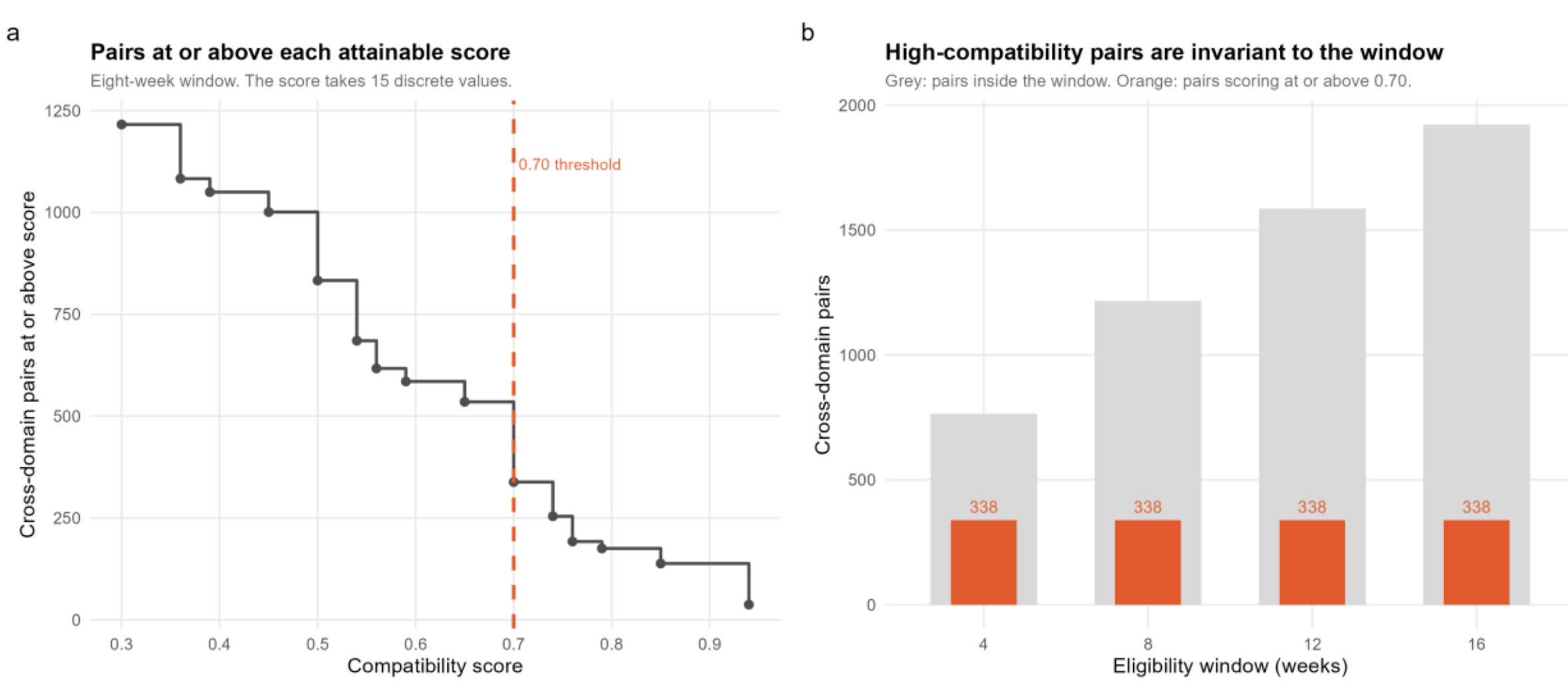

Supplemental Figure 5: Sensitivity of the campaign compatibility score (Analysis S6). a, Cross-domain pairs at or above each attainable score within the eight-week window, with the 0.70

threshold marked; the score takes 15 discrete values. b, High-compatibility pairs by eligibility window, with the scoring function held fixed: grey bars are all cross-domain pairs inside the window and orange bars those scoring at or above 0.70, which remain at 338 across every window from four to sixteen weeks.

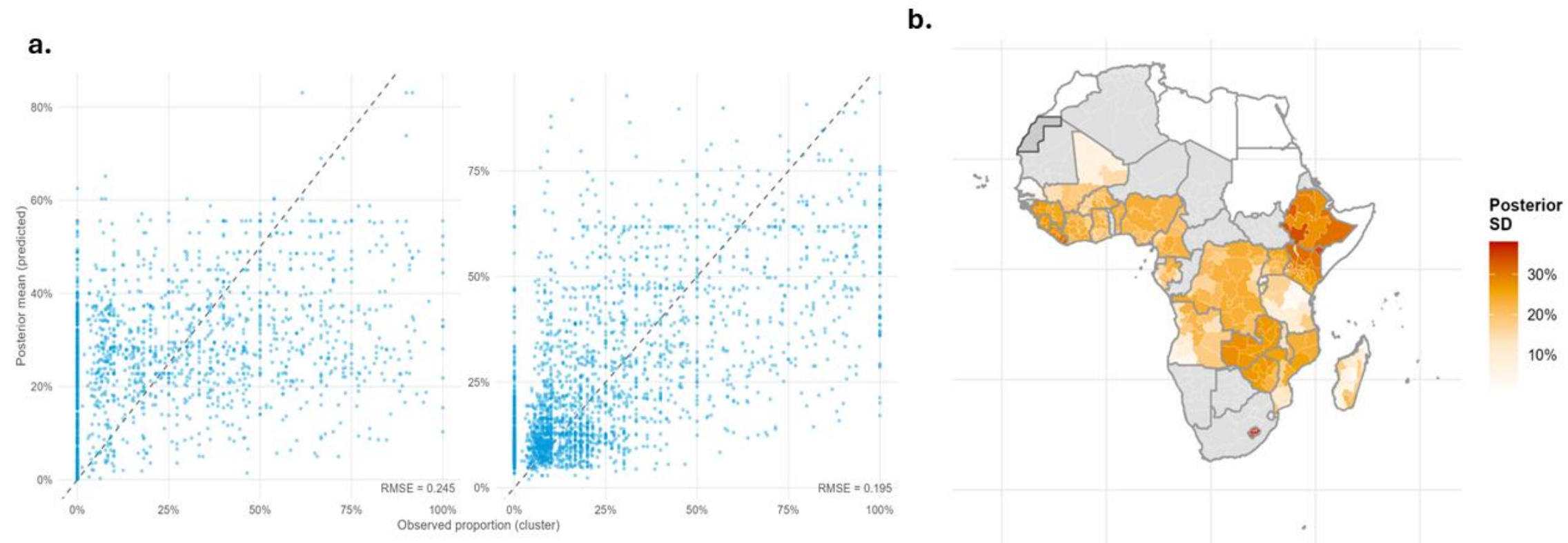

Supplemental Figure 6: Validation and posterior uncertainty of the cluster-level model. a, Observed versus predicted cluster proportions on a 20% random holdout (seed 2026) for RDT positivity (left) and zero-dose prevalence (right); dashed line is the identity, RMSE annotated. b, Posterior standard deviation of the Admin-1 RDT-positivity estimate; higher values indicate units with fewer clusters or weaker spatial borrowing.

References

1 Riebler, A., Sorbye, S. H., Simpson, D. & Rue, H. An intuitive Bayesian spatial model for disease mapping that accounts for scaling. *Statistical Methods in Medical Research* **25**, 1145-1165 (2016). https://doi.org:10.1177/0962280216660421

2 Simpson, D., Rue, H., Riebler, A., Martins, T. G. & Sorbye, S. H. Penalising model component complexity: a principled, practical approach to constructing priors. *Statistical Science* **32**, 1-28 (2017). https://doi.org:10.1214/16-STS576
